# Validation of the International Prostate Symptom Score in Chinese males and females with lower urinary tract symptoms

**DOI:** 10.1186/1477-7525-12-1

**Published:** 2014-01-02

**Authors:** Edmond PH Choi, Cindy LK Lam, Weng-Yee Chin

**Affiliations:** 1 Department of Family Medicine and Primary Care, The University of Hong Kong, 3/F., 161 Main Street, Ap Lei Chau Clinic, Pokfulam, Ap Lei Chau, Hong Kong

**Keywords:** Questionnaires, Psychometric, Lower urinary tract symptoms, Quality of life, Urinary bladder diseases

## Abstract

**Objectives:**

To evaluate the translation of the IPSS (Hong Kong Chinese version 1) and to assess the applicability, validity, reliability and sensitivity of the instrument in both males and females with LUTS in Chinese population.

**Methods:**

The translation of the IPSS (Hong Kong Chinese version 1) was reviewed through back translation. Modifications were made, resulting in the development of The IPSS (Hong Kong Chinese version 2). The content validity was assessed by contend validity index. 233 subjects with LUTS were recruited in Hong Kong primary care settings for pilot psychometric testing. The construct validity was assessed by corrected item-total correlation and Pearson’s correlation test against ICIQ-UI SF, IIQ-7 and SF-12 v2. The reliability was assessed by the internal consistency (Cronbach’s Alpha coefficient) and test –retest reliability (Intraclass correlation coefficient). The Sensitivity was determined by performing known group comparisons by independent *T*-test.

**Results:**

The content validity index for all items could reach 1. Corrected item-total correlation scores were ≥0.4 for four symptom questions (feeling of incomplete bladder emptying, intermittency, weak stream and straining). Overall, the total symptom score moderately correlated with ICIQ-UI SF. The quality of life score moderately correlated with the IIQ-7 but weakly correlated with SF-12 v2. Overall, the reliability of the IPSS (Hong Kong Chinese version 2) was acceptable (Cronbach’s Alpha coefficient = 0.71, ICC of the symptom questions =0.8, ICC of the quality of life question =0.7). The symptoms questions and quality of life questions of the IPSS (Hong Kong Chinese versions 2) were sensitive in detecting differences between groups.

**Conclusions:**

The IPSS (Hong Kong Chinese version 2) is a valid, reliable and sensitive measure to assess Chinese females and males with lower urinary tract symptoms. The IPSS quality of life question is more sensitive than the generic quality of life measure to differentiate subgroups.

## Background

Lower urinary tract symptoms (LUTS) are common, particular among the elderly. LUTS have adverse impact on health- related quality of life (HRQOL). Most of the treatments like surgery, medication and behavioral therapy aim to reduce the symptom severity and alleviate the negative impact on HRQOL. In both clinical practice and research, the symptom severity and the negative impact of LUTS should be accurately assessed and properly recorded. Patient report outcome measures usually in the form of questionnaire provide a method for the standardized collection of data from patients. The American Urological Association (AUA) symptom index is a 7-item questionnaire originally developed and validated by the AUA [1]. It asks about the severity of LUTS namely, incomplete bladder emptying, frequency of urination, intermittency, urgency, weak urine stream, straining and nocturia. Each of the questions is rated from 0 (not at all) to 5 (almost always). The total symptom score is the sum of questions 1–7. According to the total symptom score, the severity of LUTS can be graded as mild (0–7), moderate (8–19) and severe (20–35). The AUA symptom index was subsequently modified by the World Health Organization with the addition of one item on quality of life and renamed the International Prostate Symptom Score (IPSS) for assessing males with LUTS. The answers to the quality of life question range from 0 (delighted) to 6 (terrible). The IPSS has been translated into several languages and is widely used in clinical practice and research, however, only the French, Spanish, Malay, Japanese and Arabic versions have been reported to be valid and reliable for assessing males with LUTS [2-6]. Furthermore, the IPSS was originally developed for assessing LUTS in males. Several studies have also used the IPSS to assess females with LUTS [7,8]. It has been found to be a valid and reliable patient reported outcome measure for females with LUTS in Japan [9].

Translating well-established instruments into different languages, and testing its validity to prove that the attributes being measured in various settings are the same is needed to enable cross-cultural comparisons of findings. It is needed to ensure the adapted instrument is conceptually equivalent to the original version and relevant and culturally acceptable to the target population. This is known as cross-cultural adaptation. The IPSS was translated into Chinese by the Hong Kong Urological Association (HKUA) in 1995 [10] and has been used in clinical practice and research for assessing Chinese male patients with LUTS. To date however, the translation and psychometric performance of the IPSS (Hong Kong Chinese version 1) has never been examined. The objectives of this study were to evaluate the translation of the IPSS (Hong Kong Chinese version 1), to refine the translation if required, and to assess the applicability, validity, reliability and sensitivity of the instrument in both Chinese males and females with LUTS.

### Methods and subjects

#### Evaluation on the translation of the IPSS (Hong Kong Chinese version 1)

The IPSS (Hong Kong Chinese version 1) was back translated into English by a professional translator who was blind to the original IPSS. The back translation was reviewed against the original English IPSS by two bilingual authors (EPHC and CLKL) and a third author (WYC) who is a native English speaker. According to the International Society For Pharmacoeconomics and Outcomes Research (ISPOR), some constructs like medical symptoms require more literal translation whilst subjective constructs like health- related quality of life need to be more conceptually equivalent [11]. Discrepancies between the back translation and original English version were identified. Items were revised by the authors resulting in the development of the IPSS Hong Kong Chinese version 2 (IPSS HKv2).

### Content validity

Cognitive debriefing interviews on the IPSS HKv2 were subsequently conducted on ten Chinese (Cantonese) speaking patients with LUTS to assess the clarity, relevance and interpretation of each question and response option by the first author (EPHC). Subjects for the cognitive debriefing interviews were recruited by convenience sampling, balanced for age and sex, from a nurse-led continence clinic. The following questions were asked: (1) whether the subjects could understand the questions and response options, (2) to interpret what the questions and response options meant by subjects’ own wording, (3) whether the questions and response options were relevant to lower urinary tract symptoms. The answers of the interviews were recorded verbatim. Both bilingual authors (EPHC and CLKL) reviewed the results of the cognitive debriefing interviews. The IPSS HKv2 was finalized and pilot tested on males and females with urinary symptoms.

### Pilot psychometric testing of the IPSS

Subjects for pilot testing of the IPSS HKv2 were recruited from 2 different settings to include patients with a cross spectrum of disease severity. One group, patients with LUTS attending nurse-led continence clinics, were recruited by consecutive sampling. The other group, primary care patients with LUTS attending general outpatient clinics were identified by waiting room screening. The screening instrument used was adapted from the International Consultation on Incontinence Questionnaire-Urinary Incontinence Short Form (ICIQ-UI SF) using scores ≥ 3 to identify eligible subjects. The English translation of the adapted ICIQ-UI SF was shown in Additional file 1. Subjects from both settings were excluded if they were aged <18 years, could not understand Cantonese, refused to participate, or were too ill to give consent.

Eligible patients were approached by a field worker who explained the aims, procedures and nature of the study. Subjects who consented were asked to provide their contact details and were contacted within two weeks of recruitment by trained research assistants who administered the study instruments by telephone interview. Patients recruited from general outpatient clinic waiting rooms were contacted again two-weeks after their baseline interview to collect data for test-retest reliability.

A sample size of 200 subjects (100 subjects in continence clinics and 100 subjects in general outpatient clinics) was planned based on the recommendation for pilot psychometric testing [12]. This sample with 100 subjects in each group (continence clinics and general outpatient clinics) was able to detect a statistically significant difference between groups by independent *T*-test with 80% power (p = 0.05, two tailed) and a moderate Cohen’s effect size of 0.4.

Patients gave written informed consent fo the use of their data. The study was approved by the institutional review boards: HKWC (UW 12-558), HKEC (HKEC-2010-095), KWC (KW/EX/10-149(34-16)) and KEC (KC/KE-10-0209/ER-3).

### Study instruments

In addition to the IPSS HKv2 the following instruments were administered to subjects to evaluate convergent validity.

### Incontinence impact questionnaire – short form **(IIQ-7)**

The IIQ-7, which consists of 7 questions, was originally used to evaluate the impact of urinary incontinence on health -related quality of life. The psychometric properties were tested in females in Hong Kong [13]. The term “urinary leakage” was modified to “urinary problems” in the present study in order to extend the scope of application of the measure. The total score is the sum of questions 1–7. The higher the total score of the IIQ-7, the more the negative impact on health- related quality of life.

### Consultation on incontinence questionnaire-urinary incontinence short form **(ICIQ-UI SF)**

The ICIQ-UI SF consists of four questions to assess the frequency (question 1) and amount of urinary leakage (question 2), the impact of urinary leakage on quality of life (question 3) and the perceived causes of urinary leakage (question 4) [14]. The term “urinary leakage” was modified to “urinary problems” in frequency and quality of life questions to extend the scope of application of the measure. The total score is the sum of question 1–3. Higher scores indicate higher symptom severity and greater impact on health- related quality of life. The fourth question is an unscored question about the perceived caused of urinary incontinence.

### Short form 12, version 2 **(SF-12v2)**

SF-12 v2 is a generic health-related quality of life measure, which covers eight domains namely physical functioning, role limitation due to physical problems, bodily pain, general health, vitality, social functioning, role limitation due to emotional problems and mental health. SF-12 v2 can be summarized into physical and mental component summary (PCS and MCS) scores with higher scores indicating better quality of life. It has been validated for use in Hong Kong primary care patients [15].

### Global rating of change scale

The single item scale asks subjects to rate the change in his/her health since their baseline interview and was administered to subjects who participated in the two-week follow-up telephone interview for assessing test-retest reliability. Only subjects who rated no change in their health over the 2-week period were included for evaluation of test-retest reliability.

### Data analysis

The content validity index (CVI) on clarity and relevance was assessed by examining the proportion of dichotomous responses “yes” or “no”. Items with CVI ≥0.8 were considered to have good content validity [16].

Descriptive statistics, including mean, standard deviation, and percentage of floor and ceiling of scores were calculated. 15% was used as the threshold for a significant floor or ceiling effect [17].

The internal construct validity of the IPSS HKv2 was assessed by examining the corrected item- total scale correlation using corrected item-total scale correlation scores ≥0.4 to identify adequate correlation [18]. The convergent validity of the IPSS was assessed using Pearson’s correlation test against ICIQ-UI SF, IIQ-7 and SF-12 v2. It was hypothesized that IPSS HKv2 total symptom score would have a moderate correlation (correlation coefficient between 0.4 and 0.6) with ICIQ-UI SF as both measure clinical symptoms. It was hypothesized that IPSS HKv2 quality of life score would have a stronger correlation with IIQ-7 than SF-12 v2 because both IPSS HKv2 quality of life question and IIQ-7 are condition-specific measures.

The internal consistency of the IPSS was assessed by Cronbach’s alpha using cut-off scores ≥ 0.7 to indicate adequate internal consistency [19]. Test-retest reliability was assessed by examining the intra-class correlation coefficient (ICC). ICC ≥ 0.7 was used to indicate good reproducibility [17].

Sensitivity of the IPSS HKv2 was determined by performing known group comparisons of the mean total symptom and quality of life scores by independent *T*-test. It was hypothesized that patients attending nurse- led continence clinics would have higher symptom scores and poorer health-related quality of life because their condition had already been identified by a doctor and required further management. Conversely, it was hypothesized that patients recruited from general outpatient clinics would have comparatively lower IPSS HKv2 symptom and quality of life scores as these patients were identified by screening and had not sought help for their symptoms.

All psychometric properties, except sensitivity, were analyzed by gender groups and overall. All statistical analyses were performed using SPSS 20.0.

## Results

### Translation

Back-translation of the IPSS (Hong Kong Chinese version 1) revealed that all questions, aside from the item on nocturia, and seven response options were not equivalent to the original English version The IPSS (Hong Kong Chinese version 1) uses a dichotomous leading question “Have you often…” which asks for a “Yes” or “No” response, whereas, the original English IPSS uses “How *often* have you…” which asks about the frequency of the urinary symptom. The quality of life item was also problematic in the IPSS (Hong Kong Chinese version 1) as the translation did not incorporate the meaning “the rest of your life with your urinary condition”. Two of the response options for the symptom-related questions, and all five response options of the quality of life question were not equivalent to the original English version. Additional file 2 shows all non-equivalent questions and response options.

Through panel review, all nonequivalent questions and response options were modified to enhance their translational equivalence to the original English version. The panel also further refined one of the response options for the quality of life question (“unhappy”) even though back-translation was equivalent to original English version. This was done to enhance clarity and make it easier for respondents to compare the response options.

### Content validity

Ten subjects were recruited for cognitive debriefing interviews: five males, five females; age range 23–70 years (mean 54.2 years). All subjects were able to correctly interpret each item and response options. The CVI on clarity and relevance of all items reached 1.0.

### Applicability and response rates

Two hundred and third three subjects were recruited for pilot psychometric testing of the IPSS HKv2. Five subjects could not complete the questionnaire. 98% of the data was analyzable. Baseline characteristics of study subjects are shown in Table 1. The mean time taken to complete the IPSS HKv2 was 2.40 minutes. The subject recruitment flowchart is shown in Figure 1.

**Table 1:** Characteristics of subjects of pilot psychometric testing

**Characteristics**		**CCs**	**GOPCs**	**Overall**
		**n = 100**	**n = 133**	**n = 233**
1. Mean age (SD)		61.5 (11.5)	64.4 (11.2)	63.2 (11.5)
2. Age group (years, %)				
	18-65	62.0	54.9	57.9
	Above 65	38.0	45.1	42.1
3. Gender (%)				
	Male	52.0	41.4	45.9
	Female	48.0	58.6	54.1
4. Marital status (%)				
	Married	76.0	72.9	74.2
	Single	5.0	4.5	4.7
	Widower	14.0	15.8	15.0
	Divorced	5.0	6.8	6.0
5. Employment status (%)#				
	Working	42.0	27.8	33.9
	Not working	58.0	72.2	66.1
6. Household income (%)				
	<HKD20.000	63.0	61.7	62.2
	HKD20,000 or the above	29.0	21.8	24.9
	Missing	8.0	16.5	12.9
7. Smoking status (%)				
	Never	71.0	78.2	75.1
	Ex-smoker	23.0	13.5	17.6
	Current smoker	2.0	2.3	2.1
	Refused to answer	4.0	6.0	5.2
8. Drinking status (%)				
	Never	44.0	49.6	47.2
	Ex-drinker	15.0	12.0	13.3
	Current drinker	38.0	30.8	33.9
	Refused to answer	3.0	7.5	5.6

CCs: continence clinics.

GOPCs: general outpatient clinics.

n = number of subject.

#Significant difference between patients in CCs and GOPCs by chi-square test (P < 0.05).

**Figure 1:**
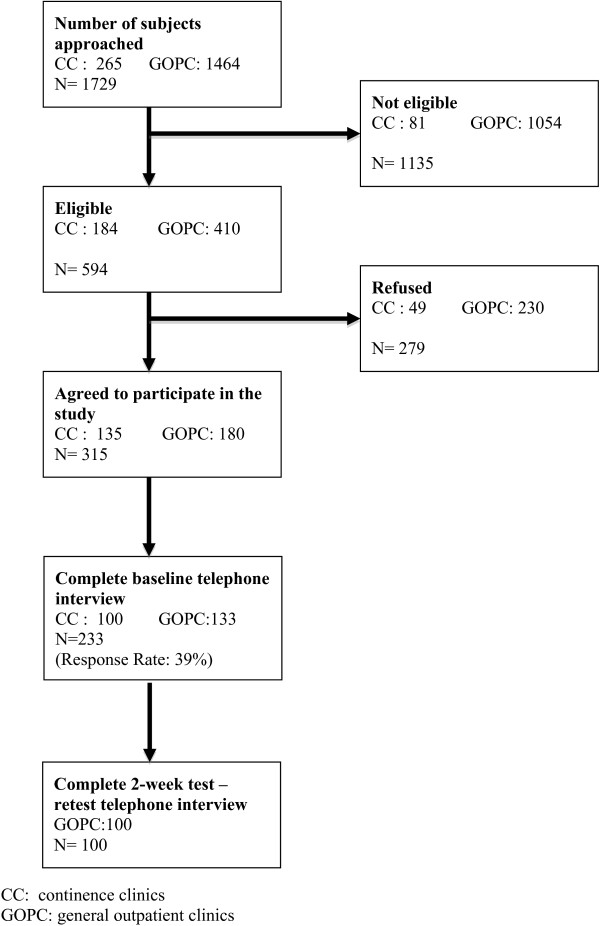
Subject recruitment flowchart.

### Psychometric performance

Table 2 shows the distribution of the responses for each item of the IPSS HKv2 by gender. For the total symptom score, 1.8% of subjects (2.4% female; 1.0% male) had the lowest possible score whilst no ceiling effect was observed. For the quality of life score, 4.7% of subjects (4.8% female; 4.7% male) had lowest possible score while 4.3% of subjects (3.2% female; 5.7% male) had highest possible score.

**Table 2:** Score distribution, corrected item-total correlation and reliability of the IPSS (HK v2) by gender

	**Female**				**Male**				**Overall**			
	**n**	**Mean**	**(SD)**	**Corrected item-total correlation**	**n**	**Mean**	**(SD)**	**Corrected item-total correlation**	**n**	**Mean**	**(SD)**	**Corrected item-total correlation**
1. Incomplete emptying	126	1.02	(1.22)	0.52	107	1.90	(1.61)	0.58	233	1.42	(1.47)	0.58
2. Frequency	126	2.01	(1.61)	0.37	106	2.37	(1.68)	0.36	232	2.17	(1.65)	0.38
3. Intermittency	126	0.79	(1.34)	0.54	107	1.41	(1.82)	0.56	233	1.07	(1.61)	0.57
4. Urgency	126	1.28	(1.60)	0.35	107	1.39	(1.67)	0.27	233	1.33	(1.63)	0.30
5. Weak stream	125	0.66	(1.22)	0.56	107	1.39	(1.56)	0.54	232	1.00	(1.43)	0.57
6. Straining	126	0.46	(1.03)	0.40	107	0.82	(1.39)	0.41	233	0.63	(1.22)	0.42
7. Nocturia	125	3.38	(1.89)	0.19	105	3.75	(1.65)	0.18	230	3.55	(1.79)	0.20
Total symptom score	124	9.50	(5.88)		104	13.03	(6.81)		228	11.11	(6.55)	
8. Quality of life	126	3.20	(1.89)		106	3.24	(1.80)		232	3.22	(1.85)	
		Female				Male				Overall		
	n	Floor	Ceiling		n	Floor	Ceiling		n	Floor	Ceiling	
Total symptom score	124	2.40%	0.00%		104	1.00%	0.00%		228	1.80%	0.00%	
8. Quality of life score	126	4.80%	3.20%		106	4.70%	5.70%		232	4.70%	4.30%	
		Female				Male				Overall		
**Cronbach’s alpha**		0.68				0.70				0.71		
		Female				Male				Overall		
**Intraclass correlation**	n				n				n			
Total symptom score	40	0.75			37	0.81			77	0.80		
Quality of life score	41	0.58			38	0.84			79	0.70		

CCs: continence clinics.

GOPCs: general outpatient clinics.

n = number of subject with valid answer.

Table 2 shows the results of corrected item-total correlation testing for the items on urinary symptoms. Corrected item-total correlation scores were ≥0.4 for four symptoms, but did not reach the standard of 0.4 for items on frequency, urgency and nocturia.

Table 2 shows the internal consistency and test-retest reliability of the IPSS HKv2. Cronbach’s alpha coefficient was 0.7 for the seven symptom-related items in males while 0.68 in females. Test-retest reliability was assessed in 77 patients whose global rating of change scale showed no change in their health between the baseline and 2-week interviews. Overall the ICC of the IPSS HKv2 total symptom and quality of life scores exceeded 0.7, and exceeded 0.8 in males. The ICC of the IPSS HKv2 quality of life question was only 0.58 in females.

Table 3 shows the results of Pearson’s correlation testing of total symptoms and quality of life scores against the ICIQ-UI SF, IIQ-7, SF-12v2 PCS and MCS scores. The IPSS HKv2 total symptom score had a moderate correlation with ICIQ-UI SF (Pearson’s correlation coefficient of 0.44 in females and 0.50 in males). The IPSS HKv2 quality of life score had a moderate correlation with IIQ-7 but weak correlations with SF-12v2 PCS and MCS. In males, there was no significant correlation between the IPSS HKv2 quality of life score and SF-12 PCS.

**Table 3:** Correlations of the IPSS (HK v2) with ICIQ-UI SF, IIQ-7, SF 12 v2

		**Pearson’s correlations**			
		**ICIQ -UI SF**	**IIQ-7**	**SF 12 PCS**	**SF 12 MCS**
Female	The IPSS total symptom score	0.44**			
	The IPSS quality of life score		0.50**	−0.22*	−0.18*
Male	The IPSS total symptom score	0.50**			
	The IPSS quality of life score		0.40**	−0.10	−0.28**
Overall	The IPSS total symptom score	0.47**			
	The IPSS quality of life score		0.46**	−0.17*	−0.21**

**Correlation is significant at the 0.01 level (2-tailed).

*Correlation is significant at the 0.05 level (2-tailed).

Table 4 shows the sensitivity of the IPSS HKv2 in detecting differences between subjects recruited from continence care clinics and those recruited from general outpatient clinic waiting rooms. As expected, statistically significant differences were detected between the two groups for IPSS HKv2 total symptom score (effect size 0.34) and quality of life scores (effect size 0.64). Similarly, significant differences were detected between the groups for ICIQ-UI SF (effect size 0.38) and IIQ-7 (effect size 0.30). There was no significant difference between groups for SF-12v2 PCS and MCS.

**Table 4:** Sensitivity in detecting differences between subjects from CC and GOPC

	**CC**		**GOPC**			
**Scale**	**Mean**	**(SD)**	**Mean**	**(SD)**	**P-value**	**ES**
The IPSS total symptom score	12.33	(6.36)	10.16	(6.56)	0.013	0.34
The IPSS quality of life score	3.86	(1.67)	2.74	(1.83)	<0.001	0.64
The ICIQ-UI SF	8.48	(3.99)	6.94	(4.01)	0.004	0.38
The IIQ-7	3.77	(4.13)	2.59	(3.75)	<0.024	0.30
The SF-12 PCS	46.46	(9.14)	46.08	(9.58)	0.764	0.04
The SF-12 MCS	52.59	(10.88)	53.44	(10.26)	0.545	0.08

CCs: continence clinics.

GOPCs: general outpatient clinics.

ES: effect size.

### Comments

Back translation of the IPSS (Hong Kong Chinese version 1) identified several questions and response options which were not equivalent to the original English instrument. First, “*have you*” instead of “*how often*” is used for every question in the Hong Kong version 1. When respondents first read the question, they might only focus on whether they have the particular symptom and may not expect to consider the frequency of the symptom, causing discrepancies in how they might respond. Second, the meaning of “*the rest of your life*” is missed in the quality of life question in the Hong Kong Chinese version 1. Its purpose in the original instrument is to ask respondents to consider how they would feel if their urinary problems lasted until they died. Without the meaning of “*the rest of your life*”, the respondent might only consider the current or short-term impact of their urinary problem. Such translational discrepancies threatens the validity of data [20], and can affect its cross-cultural interpretability. This is the first study to support the content validity of the IPSS for both male and female patients LUTS. Both males and females could understand and correctly interpret the question items and response options. It confirmed that the questions of the IPSS are not gender specific.

In item-total correlation testing, the symptom-related items on frequency, urgency and nocturia had poor correlation in females and males. These three symptoms are predominantly storage symptoms and may suggest that these items are measuring a related but slightly different domain than the other items of the IPSS HKv2.

The IPSS HKv2 total symptom score moderately correlated with the ICIQ-UI SF which confirms that the constructs of both measures are related but not equivalent. The IPSS quality of life question had a moderate correlation with IIQ-7 but weak correlation with SF-12v2 suggesting that there is a difference in the construct of the IPSS quality of life question and that of the SF-12v2. Since SF-12v2 is a generic health-related quality of life measure, the domains of such measures might not be specific and sensitive enough to capture the impact of LUTS on health-related quality of life. Generic measures contain irrelevant domains and may miss specific concerns held by the respondents [21]. On the contrary, the IIQ-7 is a condition-specific measure, so the domains of IIQ-7 should be more relevant to those with LUTS. Our results support the added value of condition-specific measures.

Interestingly, the IPSS HKv2 quality of life score only correlated with SF-12v2 MCS in men whilst there is a stronger correlation between IPSS HKv2 quality of life score and SF-12v2 PCS in women. This shows that the impact of LUTS on quality of life is different for males and females. The present study showed that LUTS appears to have a greater impact on mental health than physical health in men, whereas the opposite occurs in women. These findings are consistent with the validation study of the Spanish IPSS [3]. Males with LUTS are worried and embarrassed [22], and have concerns about sexual competence and prostate cancer [23]. Men with LUTS might therefore have more mental health burden. Conversely, in women, overactive symptoms such as urgency and frequency appear to have a more negative impact on physical functioning than mental health [24,25].

The IPSS HKv2 was found to be a reliable patient reported outcome measure in both males and females. Overall, the internal consistency (Cronbach’s alpha >0.7) of the IPSS HKv2 appears to be comparable to the original English instrument [1]. The Cronbach’s alpha was slightly below standard in females, which is still acceptable. The 2-week test-retest reliability in patients with stable health condition was acceptable and comparable to other versions of the IPSS [1,3,26]. The intra-class correlation coefficients of IPSS HKv2 total symptom and quality of life scores were above 0.7 with the exception of the quality of life question in female subjects (ICC = 0.578) implying that this single-item measure is comparatively unreliable in women [12]. Clinicians and researchers should interpret the responses to this item with caution and should not rely on the IPSS single-item quality of life question to monitor patients longitudinally or for evaluation of treatment effect.

The finding of higher IPSS HKv2 total symptom severity and poorer health-related quality of life in patients recruited from continence clinics than general outpatient clinics shows that the instrument is sensitive to differentiate patients with varying disease severity. It should be noted that the IPSS quality of life question had a larger effect size than all the other measures suggesting that this single-item measure may have high sensitivity to differentiate subgroups. Our findings also suggest that an individual’s perceived impact on health-related quality of life might be a determinant for patients to seek medical advice. From our data, it appears that the SF-12v2 may not be sensitive enough to detect differences between patients in continence clinics and general outpatient clinics. The SF-12v2 may be too generic and not sufficiently sensitive to detect subgroup differences [27,28]. Our findings indicate that condition-specific measures outperform generic measures for detecting differences between subgroups of patients with urinary symptoms.

### Limitations

The subjects were recruited in the government-funded primary care setting by convenient sampling. Patients in private primary care setting or in secondary care were not included in the pilot psychometric testing. The responsiveness of the instrument (its ability to detect change over time) still needs to be assessed. Subjects in this study were mainly Cantonese speakers and the content validity and psychometric performance of the instrument should be further tested with Mandarin speakers or Chinese speakers of different nationalities.

## Conclusions

The IPSS Hong Kong Chinese version 2 (IPSS HKv2) is both conceptually and linguistically equivalent to the original English IPSS and the present study has shown its applicability, content validity, construct validity, reliability and sensitivity. The IPSS quality of life question is sensitive than generic health- related quality of life measure to differentiate subgroups. We hope the measure can be applied for Chinese in the worldwide diaspora.

## Abbreviations

AUA: American Urological Association; CC: Nurse-led continence clinic; CVI: Content validity index; GOPC: General outpatient clinic; HKUA: Hong Kong Urological Association; HRQOL: Health- related quality of life; ICC: Intra-class correlation coefficient; ICIQ-UI SF: International Consultation on Incontinence Questionnaire -Urinary Incontinence-Short Form; IIQ- 7: Incontinence impact questionnaire-7; IPSS: International prostate symptom score; IPSS HKv2: International prostate symptom score (Hong Kong Chinese version 2); LUTS: Lower urinary tract symptoms; MCS: Mental component summary; PCS: Physical component summary; SD: Standard deviation; SF-12 v2: Short Form-12 version 2; SPSS: Statistical package for the social science.

## Competing interests

We declare there are no conflicts of interest specific to this manuscript. The research study was supported by the Small Project Fund, The University of Hong Kong.

## Authors’ contributions

Mr. PH C is responsible for study design, acquisition of data, data analysis, drafting of the manuscript. Dr. WY C and Prof. CLK L are responsible for study design, supervision, critical revision of the manuscript and obtaining funding. All authors read and approved the final manuscript.

## Supplementary Material

Additional file 1 Screening questionnaire (adapted ICIQ-UI SF). Click here for file

Additional file 2 Nonequivalent questions and response options of the IPSS (Hong Kong version 2). Click here for file
