# Supplementary material for: Validation of the International Prostate Symptom Score in Chinese males and females with lower urinary tract symptoms
Source: Health Qual Life Outcomes. 2014 Jan 2;12:1. doi: 10.1186/1477-7525-12-1 (PMC3883473; doi:10.1186/1477-7525-12-1)
Supplement: Additional file 1 — Screening questionnaire (adapted ICIQ-UI SF). [file 1477-7525-12-1-S1.docx]

**Additional file 1: Screening questionnaire (adapted ICIQ-UI SF)**

1. How often do you have urinary problems *?

Never☐ 0
About once a week or less often ☐ 1
Two or three times a week ☐ 2
About once a day ☐ 3
Several times a day ☐ 4
All the time☐ 5

2. We would like to know how much urine you think leaks.
How much urine do you usually leak (whether you wear protection or not)?

None ☐ 0
A small amount ☐ 2
A moderate amount ☐ 4
A large amount ☐ 6

3. Overall, how much do urinary problems * interfere with your everyday life?
Please ring a number between 0 (not at all) and 10 (a great deal)

0 1 2 3 4 5 6 7 8 9 10

Total Score: 1+2+3:____

*Urinary problems: urinary incontinence, incomplete emptying, dribbling, difficult urination, frequency of urination
