# Supplementary material for: Validation of the International Prostate Symptom Score in Chinese males and females with lower urinary tract symptoms
Source: Health Qual Life Outcomes. 2014 Jan 2;12:1. doi: 10.1186/1477-7525-12-1 (PMC3883473; doi:10.1186/1477-7525-12-1)
Supplement: Additional file 2 — Nonequivalent questions and response options of the IPSS (Hong Kong version 2). [file 1477-7525-12-1-S2.docx]

**Additional file 2.** Nonequivalent questions and response options of the IPSS (Hong Kong version 2)

| **Original English version** | **Back-translation of Hong Kong Chinese version 1** |
| --- | --- |
| During the last month or so, how often have you had a sensation of not emptying your bladder completely after you finished urinating? | In the past month, have you often felt unable to empty your bladder completely when urinating? |
| During the last month or so, how often have you had to urinate again less than two hours after you finished urinating? | Over the past month, have you often needed to urinate more than once every two hours? |
| During the last month or so, how often have you found you stopped and started again several times when you urinated? | Over the past month, has it been common for your urine stream to stop and start? |
| During the last month or so, how often have you found it difficult to postpone urination? | Over the past month, have you often found it difficult to suppress urination? |
| During the last month or so, how often have you had a weak urinary stream? | Over the past month, have you often experienced a weak urine stream? |
| During the last month or so how often have you had to push or strain to begin urination? | Over the past month, has it often taken you a great effort before you start urinating? |
| If you were to spend the rest of your life with your urinary condition just the way it is now, how would you feel about that? | Considering your current situation in terms of passing urine, how would you rate your quality of life henceforth? |
| Less than 1 time in 5 | Less than one fifth of the time |
| 5 or more times | More than five times |
| Delighted | Very good |
| Pleased | Good |
| Mostly satisfied | Satisfactory |
| Mixed (about equally satisfied and dissatisfied | Okay |
| Mostly dissatisfied | Unsatisfactory |
